# Gatekeepers of health: A qualitative assessment of child care centre staff's perspectives, practices and challenges to enteric illness prevention and management in child care centres

**DOI:** 10.1186/1471-2458-8-212

**Published:** 2008-06-13

**Authors:** Marsha Taylor, Cindy L Adams, Andrea Ellis

**Affiliations:** 1 Department of Population Medicine, Ontario Veterinary College, University of Guelph. 50 Stone Rd. E, Guelph, Ontario, N1G 2W1, Canada; 2 Foodborne, Waterborne and Zoonotic Infections Division, Public Health Agency of Canada. 255 Woodlawn Rd. W, Unit 120, Guelph, Ontario, N1H 8J1, Canada; 3 Faculty of Veterinary Medicine, University of Calgary, 3330 Hospital Drive NW, Calgary, Alberta, T2N 4N1, Canada

## Abstract

**Background:**

Enteric outbreaks associated with child care centres (CCC) have been well documented internationally and in Canada. The current literature focuses on identifying potential risk factors for introduction and transmission of enteric disease, but does not examine why these risk factors happen, how the risk is understood and managed by the staff of CCCs, or what challenges they experience responding to enteric illness. The purpose of this study was to explore the understanding, knowledge and actions of CCC staff regarding enteric illness and outbreaks, and to identify challenges that staff encounter while managing them.

**Methods:**

Focus groups were conducted with staff of regulated CCCs in Southern Ontario. Five focus groups were held with 40 participants. An open ended style of interviewing was used. Data were analyzed using content analysis.

**Results:**

CCC staff play an important role in preventing and managing enteric illness. Staff used in-depth knowledge of the children, the centre and their personal experiences to assist in making decisions related to enteric illness. The decisions and actions may differ from guidance provided by public health officials, particularly when faced with challenges related to time, money, staffing and parents.

**Conclusion:**

CCC staff relied on experience and judgment in coordination with public health information to assist decision-making in the management of enteric illness and outbreaks. Advice and guidance from public health officials to CCC staff needs to be consistent yet flexible so that it may be adapted in a variety of situations and meet regulatory and public health requirements.

## Background

Research states that early childhood education and care is important for healthy child development [1], and public health can be a primary partner and supporter in the delivery of early childhood programs in the community [2].

In 2003, over 2 million Canadian children from the ages of 0–5 years were in some form of non-parental care and 28% of these children were in a daycare [3]. The cost of child care ranged from $300 to over $700/week and varied between provinces, territories and age in 2005 [4]. Two types of child care, regulated and unregulated, are present in Canada. There are approximately 11, 000 regulated child care facilities in Ontario which include nursery schools, preschools, centre-based full-day child care and regulated home child care. These centres must meet legislated requirements for operation of services as set out by provincial/territorial regulations. Unregulated arrangements may consist of care provided by a relative, by an unregulated family child care provider, or in-home caregiver [5].

Canadian data from 1998 showed that over 98% of child care centre (CCC) staff were female and 45% were under the age of 30. Staff had a high level of experience; 60% had worked in the field over 5 years and 71% of teaching staff held an early childhood education (ECE) credential. Hourly wages varied between provinces and territories. The national average hourly rate was $11.62 for a teacher and $9.59 for an assistant. Only 74% of staff reported paid sick days, which averaged 7.6 days/year. Even with these challenges, a high proportion of staff reported experiencing enjoyment and reward from their work. Twenty percent reported feeling disrespected by some other professionals [5].

Numerous enteric illness outbreaks in CCCs have been reported, although investigations rarely identified a common source. Reports commonly cited person-to-person transmission or risk factors that increased the potential for transmission and illness [6-8]. These factors included inadequate hygiene related to diapering, toileting and hand-washing [9,10]; poor cleaning of environmental surfaces [6]; improper food preparation [6]; inadequate exclusion of ill children and staff [9,10]; and working or being cared for in a younger age group [7,11].

Acts and regulations related to child care and health are issued by the provinces and territories in Canada. Additionally, public health officials at the local level have a high level of responsibility for performing inspections, responding to outbreaks and providing guidance or developing resources for CCCs according to these Acts and regulations. Staff of CCCs are most likely made aware of these Acts and regulations through their formal training, but also through regular interaction with public health officials. The variation between provincial/territorial or local health regions can make consistent public health response to enteric outbreaks challenging. Currently, knowledge is limited on how staff working in CCCs interpret and implement the guidance they are provided from public health officials. A previous study identified that staff may have inconsistent definitions of what constitutes diarrhea, and that their actions during an outbreak may differ from those recommended by public health officials [11]. Furthermore, reports from three recent Canadian investigations recommended the development of consistent guidance for CCCs related to outbreak management, prevention and control of illness [9,11,12].

Resources or programs can be more successful when developed in collaboration with the groups or people who may be impacted by them [13]. Staff of CCCs were identified as an important group to consult with regarding the development of consistent guidance. The exploration of perspectives, understanding and opinions were important aspects, and thus qualitative research approaches were selected as the most suitable tool to gather this information. The qualitative approach allowed for data to be collected in the form of the participant's own words which provided a more detailed and deeper understanding than would be possible through quantitative studies. Use of qualitative methods required an inductive approach to explore the participant's experiences and perspectives [14]. Focus groups are a mechanism for collecting data. This form of group interview sets up an environment for the participants to interact, and interaction can lead to a wide range of information on experiences and perspectives [15].

The purpose of this study was to explore the perspectives, knowledge, self-described practices and challenges CCC staff working in regulated centres in southern Ontario have related to enteric illness and outbreaks. It was also intended to investigate how staff use current public health guidance. The final objective was to gather information to feed into the development of consistent recommendations for the prevention and management of enteric illness in child care settings.

## Methods

### Study Design

Between November 2005 and January 2006, five focus groups were conducted with staff from regulated CCCs in Southern Ontario. Four groups were conducted at different CCCs. Each of the four groups consisted of staff from the respective CCCs. The fifth group was mixed and consisted of staff from different centres and who did not work together. Ethics approval was obtained from the Research Ethics Board of the University of Guelph. All participants provided written, informed consent

### Study Participants

A purposive sampling approach was used to recruit participants from regulated CCCs in southern Ontario. Centres were selected by the researchers and contacted by phone. Study information and requirements were sent to each centre for their further consideration and permission to conduct the focus groups. This sampling approach sought participants who worked in a variety of settings and who could contribute their experiences on managing enteric outbreaks. Centres and staff members were defined as those who provided daytime care for an entire day to a group of children (e.g. did not include those who provided only after-school care), and whose staff's first language was English. Participating staff had daily direct contact with the children and had worked in a centre for more than six months.

### Data Collection and Analysis

Preliminary research was conducted with public health officials and CCC staff to develop appropriate questions for use in the focus groups. One-on-one interviews were conducted with public health officials to gather information related to the types of centres that they worked with, how enteric illness outbreaks were typically managed, what support public health officials provided, and their experiences while working with CCC staff. Observations of daily operations were also conducted in five centres to better understand the working environment and how staff interacted with their coworkers and supervisors. Time spent observing in the centres ranged from two hours to a full day.

Open ended inquiry was used in each focus group. Sixteen questions were developed and used per session. Questions related to staff priorities, knowledge of enteric illness in the CCC, definitions and practices, how they used information from public health officials, challenges they faced when managing enteric illness and recommendations for improvement of these situations.

Public health officials assisted in the verification of the question set prior to the focus groups to ensure clarity of the order, timing and flow of discussion. The inductive process used during and between groups with the CCC staff allowed the questions to be altered or refined. For example, follow-up or probing questions were added to allow for experiences and details not in the original question set to be captured as directed by the participants. This process of modifying questions ensured that new situations and recurring trends were explored thoroughly. Focus groups were conducted in a CCC or public location and lasted between 45 and 60 minutes. The principal investigator moderated all groups and a co-facilitator assisted by taking detailed notes. All groups were audio-taped using a digital recorder and transcribed verbatim by the principal investigator. Additionally, detailed memos were written by the researcher following each group. Notes pertained to group participation, how questions were responded to, interesting features of the discussion, and considerations for future groups.

Content analysis was used to analyze the data. This involved examining the transcripts and use of descriptive codes to compare and look for common patterns and themes among the groups [14]. All transcripts were examined by the principal investigator for common patterns within groups. Next, transcripts were reviewed and coded using descriptive codes such as: "communication with parents", "description of diarrhea" and "acknowledgement". Further content and theme analysis was done using NVivo 7. NVivo is a software program often used by qualitative researchers to handle text based data. It assists with the categorization, analysis and displaying of data [16]. Descriptive codes were later grouped into larger categories. Through continuous comparison of the transcripts, categories that were common among the groups were refined. This constant comparison allowed for the identification of themes based on common categories among the groups to be identified.

## Results

A total of 40 staff participated in one of five focus groups. The number of participants per group ranged from 3–12. All participants were female and the median age was 35 years (range 20–60 years). Eighty-three percent of the participants had an ECE Diploma (college), and 15 % had a University degree. The median number of years working in a child care centre was 11 (range 2–30 years). The centres represented a variety of settings from large, public centres with multiple classes for infants, toddlers, preschool and school-aged children to smaller centres which only had two rooms. The centres also differed on geographic location and the type of population they served.

Five major themes emerged from the data. The following is a description of the themes and text based examples to illustrate them.

### Eyes and Ears: Tools for Surveillance

Among the groups, the health and safety of the children was the primary concern and responsibility of the staff. Staff ensured health and safety through observation and the creation of an informal surveillance system for any illness among the children.

Staff: *You're concerned with the child, the last thing on your mind is really what is happening at the time,...you're concerned about what's the next step, I'm taking care of this child.*

Staff reported an intimate knowledge of the behaviour and health status of each child they cared for, including indicators of enteric illness such as bowel movements. Their intimate understanding of the children was used to monitor and look for cues that indicated a change in the child's health. These cues included changes in behavior, eating or sleeping patterns, additional symptoms as well as a change in the frequency, colour and consistency of a child's bowel movement.

Staff: *You get to know the smell, like the regular smell of somebody's bowel movement, the colour, you get to know the timing, like something is different or not right.*

Staff recognized that bowel movements, and the causes of irregular bowel movements, vary between children and took these factors into account when making decisions and taking actions. This knowledge allowed them to consider additional factors such as the use of antibiotics and medication, teething, nutritional intake as well as the period of time that would most likely indicate an infectious disease as the cause of illness.

Staff:*...and that's the hardest thing..., is this really diarrhea from a virus or is it just they're on antibiotics or they're teething.*

Staff worked in a dynamic and continually changing environment. Based on previous experience, staff knew when enteric illness appeared infectious, for example, if they observed multiple children ill throughout the centre with similar symptoms in a short period of time. They also noted that illness was often first identified in younger ages and spread throughout the centre rapidly.

Staff: *The number of cases of anything, you know when you start to see so many children with similar symptoms-quickly in succession that's when you start wondering if something is happening.*

Staff monitored the health and safety of the children by collecting information on various forms (attendance, toileting, health, etc.) and in personal journals. Staff described collecting a large amount of information, however, the number and types of forms being used, the detail collected and how the information was recorded and shared varied between centres. There was no standardardization of forms, although in some jurisdictions standard forms may have been recommended by public health officials. Information collected by staff often remained specific to the room or the group of children that they worked with. The supervisor or director of the entire centre was responsible to collect it centrally and track the information. Staff relied on the supervisor to identify centre-wide health concerns and inform all staff, as well as act as a liaison with public health.

Staff: *So the supervisor's the sounding board, all the information goes to her and then decisions are made from there.*

Monitoring and observation was reduced when a child was at home or in school, wherein the staff were unable to monitor hand-washing or health status and had to rely on parents and teachers for information. The staff had less ability to monitor older children (closer to kindergarten-age), as the child's independence was much higher. For older children, staff were less involved in diapering and toileting and less in depth records were kept.

The staff role as "eyes and ears" created an informal surveillance system for enteric illness within the centre and was also the first stage in a decision-making process. Staff demonstrated knowledge of the children they worked with and the potential causes and symptoms of enteric illness through various cues. The system relied on the staff's recognition of symptoms and their ability to report this information for tracking and communication purposes.

### First Response and Action-oriented

In order to maintain the health and safety of the children, staff acted practically and responses were action-oriented. When a child was ill, their first actions were to care for the child.

Staff 1: *Change the diaper. Deal with it.*

Staff 2: *Get out the gloves, the pads, the wet cloths, the concentrated cleaning, clean that off.*

These practical actions ensured a safe environment for the children through thorough cleaning, hand-washing, and restricting play areas (communal water and sand play) where the potential for transmission of illness from child to child existed.

During an outbreak, staff remained action-oriented but began a more intensive cleaning procedure. Responses to outbreaks were described:

Staff: *It's just a lot cleaner in the sense that you Virox [an accelerated hydrogen peroxide cleaning agent often used as a disinfectant and for sanitation purposes] more often and more often and more often. Where normally we would do it [clean] twice a day with Virox, it's four times a day or five times a day, but I don't think it changes the actual program.*

Staff spoke easily and frequently about they type of cleaning they did during an outbreak, how often they did it, and how it became a habit that they integrated into their day.

Staff: *I mean you end up putting it into your program, you end up putting it into the transitions of your day. Like in the infants [group] for example, once one staff goes outside with the children that are awake, the other staff stays inside with the children that are sleeping and they're washing toys, it just becomes a daily part, an everyday practice.*

Staff had a high level of comfort with this response and it was equivalent to practices that would be recommended by public health officials. From their experience working in the centre, staff felt that cleaning stopped further transmission of illness. This assisted them in meeting their goals of a healthy and safe environment for the children.

### Definition Dilemma

When staff defined diarrhea, they often used visual and sensory based descriptions like: "uncontained", "out of the diaper", "running down their legs". Staff used these cues to take action. However, staff definitions or descriptions of diarrhea were not the same; there was a level of ambiguity and uncertainty and they experienced a dilemma in determining whether a child was ill or not. The staff described two reasons for this uncertainty:

1) Diarrhea could be child specific. One definition may not apply to everyone.

2) The definition of diarrhea could vary between staff members and often was considered a "judgment call".

Staff experienced a similar difficulty in clearly defining an "outbreak". In public health, an outbreak is often defined as a sudden or unexpected increase of disease within a population. [17] Staff discussed that there were a number of ways in which they determined when an outbreak was occurring. These included: increased number of children who were ill, comparison to a set baseline, more illness when compared to previous years, or situations where there were multiple illnesses.

Staff: *But there's centres that are confused about that, you know I thought it was always 10% and you might talk to one public health person and she may say, oh, it's 10%, you might talk to another one who might say go on past history.*

Staff stated very clearly their need for standard definitions for both diarrhea and outbreak to assist them when managing enteric illness. They felt that if the definitions were clear, they would know which actions to take and when.

Staff: *It's diarrhea, even to recognize diarrhea versus a loose bowel movement, cause they're two different things, that would help everybody, this is where we need experts to tell us what is the true definition of diarrhea.*

In these situations staff used their experience working in the centre, knowledge of the children and personal judgment to enable them to develop definitions that assisted them, but expressed that further guidance from public health officials would be welcomed.

### Using Experience to Respond

Staff expressed that at times they felt overloaded with demands and/or had limited resources to respond appropriately to unpredictable situations of enteric illness. In these situations, they required flexibility in how they provided care and what actions they took. Staff discussed their use of judgment and previous experience in consideration with guidance provided by public health officials to make appropriate decisions.

Staff: *From our perspective...when you get a document from any public health or outside agency they tell you what you should do, but in real life is what they say what we would do? Cause we're hands on in the field.*

Staff had different forms of education and varying experiences. This attribute influenced how staff worked with children and managed enteric illness and outbreaks. Although experience could assist in making judgments, experienced staff indicated their personal judgments of diarrhea and how they responded to situations varied. This was in part due to the uncertainty associated with how to define diarrhea. New or inexperienced staff required training by more experienced staff to assist with decision-making.

Staff: *But even I think staff still do have that difficulty ...everyone's view of diarrhea can be very different, like I've had staff call me in and say do you think this is diarrhea and I've had a look at it and said no, it's just pasty, so I think some people really have different views of what diarrhea is.*

Although policies and guidance related to management of enteric illness are provided to the centre as a whole, staff reported that modification took place on a situation specific basis. This was reported by staff when they felt that the procedures and guidelines were inflexible or differed from a situation that they experienced. In some cases, staff actions varied from what they knew, leaving the individual staff member with a great deal of responsibility in deciding what to respond to and when.

Staff: *We have to say that this is our policy, like there are certain things that we can bend on, like if the fever is 99 degrees and our policy is whatever, then yes- bring your child in. But when it's something like that [excluding a child with diarrhea], it's "this is our 24 hour policy".*

Guidance provided to staff from public health officials and CCC management in this dynamic environment needed to be flexible and pragmatic. Staff used the recommended information provided by public health officials, but also felt that they had adequate ability to modify it to unique situations. This created new practices at the frontline level that were often more specific to the situation and the centre. This responsibility, and the decisions staff made, could significantly influence the management of enteric illness and the potential for outbreaks.

### Conflict in Care (caused by challenges)

In certain situations, staff experienced conflict in the care they provided due to a specific challenge. These challenges prevented them from taking required actions, and fell into four areas: money, time, staffing and parents.

#### Money

Most centres identified that on a regular basis they used bleach and water to clean and disinfect. During outbreaks or increased illness one of the first precautions was to change to a more powerful cleaning product. Use of these products was considered important for making the environment safe for the children.

Staff: *We tend to be a little proactive, well just yesterday we had one case of diarrhea and today we sent a child home with vomiting, so that room has now been totally disinfected with Virox.*

Many staff reported that when the outbreak was considered to be over, centres returned to normal bleach and water solution due to the higher cost associated with using alternate cleaning products on a regular basis even though they felt the use of the product was beneficial.

Staff: *I was going to say that's truly the reason why, the cost, bleach is a lot cheaper than Virox.*

#### Time

Staff described that they were always pressed for time, and in certain circumstances, additional responsibilities could require changes to routines in child care that may not be feasible. The expectations to keep the environment clean, especially during an outbreak, are very high and staff felt increased pressure to find ways to incorporate this demand into an already busy schedule and maintain a high level of care.

Staff: *I mean it's a lot, on top of all the stuff with parents and the kids and keeping everyone healthy ... during outbreaks we are disinfecting every surface, every toy, like every half day to every day ... cause not only are you trying to run your usual program and everything else but you're also trying to take time within those hours to care for sick kids and do this mass disinfecting...it's just added stress and workload.*

Staff worked hard to provide the cleanest and safest environment possible, but recognized they were limited by time and resources. Cleaning products which are fast acting and can be used around the children throughout the day were deemed as necessary. Staff also often suggested that having additional staff support just to clean would be high on their wish list.

Record keeping was cited as an important component of routine monitoring, and especially so during an outbreak. Staff used several forms related to the group they cared for (e.g. attendance) and each individual child (e.g. bowel movement records). This record-keeping required a large amount of time and may prevent staff from performing other actions such as cleaning, speaking with a parent, planning other programs and activities, or looking after themselves. Forms related to enteric illness were only some of the records staff kept. Staff were also responsible for documenting information related to diet, allergens, what activities the child participated in, or behaviour they exhibited.

#### Staffing

Staff said that it was difficult for them to take time off from work when they were sick. Reasons included not having compensated sick days, concern over loss of pay, and the inability to find a substitute staff member. Staff knew that they should, and were expected to take time off when experiencing symptoms of enteric illness. For most of the staff, this could be a challenge due to a limited incentive to remain at home while ill, and they admitted not always excluding themselves.

When staff were ill, they relied on a substitute staff member to replace them. However, if a substitute staff member worked in a centre during an outbreak they were not permitted to work in another centre concurrently. This policy is in place to prevent disease transmission. Staff reported that when they are unable to find a substitute staff, they may choose to work.

Staff: *If she happens to come to my centre and then the next day we were officially in an outbreak, I would have to call that supply teacher and say "you know what, you were here yesterday and we're in an outbreak". She can come back to my centre but she can't go to [another] centre, so that's the frustrating part.*

Appropriate ratios between staff and children are regulated and must be maintained in a centre at all times. When staff became ill and were unable to work, or when an ill child needed to be segregated from the larger group until a parent could come and pick them up, the maintenance of ratios between staff and children could be compromised. In these circumstances, there may not be enough staff to segregate the child from the rest of the group. Staff balanced variables such as how ill the child was, how long they had already been in the centre, and the number of available staff, before proceeding to make an informed decision regarding how to appropriately exclude children while maintaining required ratios.

Staff 1: *Depends on availability, [of staff]...if it's a case of something like diarrhea or vomiting, we try to keep them on their own, or if we can we send them to the supervisor or keep them away from the other kids. If all the kids are going outside then maybe we keep them [inside]. We do our best to keep them separate but then in the same thought they've already spent the last three hours with these kids, it's like another 20 minutes, so sometimes we just have to say for ratios and safety sake, you're [the child] staying with us.*

Staff 2: *sometimes we just don't have the staff or ability to segregate a child.*

#### Wildcard-Parents of children in care

The relationship between staff and parents was described as positive. During an outbreak, or when a child needed to be excluded, it could become strained. Lack of understanding and level of parental awareness about the transmission of enteric illness created a barrier with staff. Some parents did not understand the importance of keeping their children at home when ill. Staff felt that experience working in the CCC gave them a greater understanding of the severity of symptoms, potential causes for illness, and when a child was truly ill due to infectious diseases.

Staff: *Parents understanding even how easily it spreads and why we need this done...because I think sometimes and understandably they're balancing a job and it's a bit of an annoyance for them.*

Staff: *That is probably one of the biggest reasons as well as being inconvenient, perhaps inconvenience is the first one...just the financial end of it is another reason why parents are perhaps tentative to withdraw their child from daycare..."we paid for that".*

Staff recognized their inability to monitor the child and ensure proper hygiene when they were sent home.

They relied on the parent's honesty regarding their child's health status.

Staff 1: *I mean the hard part with it is we are relying on parents to be completely honest.*

Staff 2: *And that [diarrhea] is a tough one to hide.*

Staff 1: *But if they come back and say, "oh no we haven't had any [diarrhea] in 48 hours", when in fact it's only been 24 hours...or whatever, then we're relying on [the parents] to share the information and trust that it's going to be truthful.*

A number of reasons why they felt parents found it difficult to come and pick up an ill child or keep them at home was discussed. These reasons included a lack of access to alternate care providers or the inability to take time off for financial reasons or the nature of the work place. During the focus groups, a number of the staff expressed empathy for the parents' situations, and staff often felt conflicted between balancing the needs of the parents and what they deemed best for the centre. They expressed that overcoming the challenges associated with parents would be the most helpful to assist them when dealing with enteric illness.

Staff: *If there was a magical way we could communicate with parents that would be great...a daycare is just about a safe place for the child, what goes on there sometimes they don't really know. They don't understand an outbreak, and why they have to take their kid home... [to] be able to educate them quicker, easier, I think would help the whole process.*

## Discussion

This study illustrated that CCC staff had an intimate knowledge of symptoms and potential causes of illness in the children they cared for. This knowledge was used in addition to experience related to enteric illness to guide their decisions and actions. An informal surveillance system to identify illness and take appropriate actions was created using the knowledge gained from daily interaction with the children and the observations and records staff kept.

Staff described a high level of comfort in their ability to thoroughly clean, and thereby to help prevent and manage enteric illness within the centre. When staff felt uncertain, they relied on their own judgment and experience to assist them. This was apparent when staff described their difficulty in defining both "diarrhea" and "outbreak". In situations where challenges related to money, time, staffing and parents were identified, or staff required flexibility in their response, staff adapted their actions to ensure appropriate care, even if it meant modifying recommendations provided by public health officials. Staff gave examples including: adapting cleaning schedules, exclusion guidance, and record-keeping. Public health officials provide guidance based on legislated regulations. The purpose of this guidance is to ensure that staff consistently achieve outcomes which protect the safety and health of the children. This includes the prompt identification of cases and outbreaks of enteric illness so that appropriate public health preventative measures can be put in place. Findings demonstrated the health and safety of the children was a priority for CCC staff but how objectives related to this priority were achieved and how guidance from public health officials was used may vary by staff and facility.

To the best of our knowledge, a study of this nature has not been done. However, the findings support other work that demonstrated staff may have inconsistent definitions and actions related to enteric illness [11]. The challenges identified, such as time, understanding and financial or logistical needs have also been documented in other studies that have examined health care practitioners and their challenges with hygiene [18-20]. Additionally, the findings help to support the need for consistent forms of management for enteric illness and outbreaks in CCCs. Consistent guidance was supported by the staff who discussed their needs related to standard definitions and actions to take based on the definitions.

The use of focus groups with staff of CCCs allowed for the collection of in-depth data about a wide range of experiences and opinions related to enteric illness and outbreaks. Development of the original question set using information collected from public health officials and staff of CCCs ensured thorough exploration of themes and experiences related to the management of enteric illness. The inductive process allowed for new questions and details to be explored, and led to a range of discussion about the perspectives, experiences and challenges of CCC staff that would not have been possible through the use of a standard, closed questionnaire. Using a purposive sampling approach ensured that participating centres represented a range of care settings available in Southern Ontario. Centres varied in the number and age of children cared for, and setting type (teaching facility, private, public, etc). Although there were some differences between staff and centres, the major themes, experiences and perspectives that the staff spoke of were the same regardless of what type of centre they worked in. Non-regulated facilities were not approached due to the challenges associated with identifying them, size and physical setting. The insight and understanding of CCC staff can be used in further development and implementation of practical guidance.

The process of developing, following and adapting policies at the frontline level has been examined in other public service workers. "Street Level Bureaucrats" are defined as public service workers who interact directly with citizens in the course of their job, and who have substantial discretion in the application of policies in the execution of their work [21]. This level of discretion and ability of the frontline worker to modify set government policies for the individual situation has also been described among police officers, social workers and others who work within the public sector [21]. Additionally, studies of nurses have demonstrated similar reliance on previous experience and visual cues such as touching, observing, listening, feeling or sensing, and "knowing" in decision-making [22,23]. Conflict in care has also been reported in health care workers who demonstrated balancing the risks and demands of caring for patients [20]. The results of our study demonstrated that the decision making process and demands placed on CCC staff are similar to those experienced by other professionals, who work in high stress environments and care for others on a daily basis.

The decision-making process that CCC staff used could best be described as Naturalistic. Naturalistic decision-making is most often associated with proficient decision-makers who have extensive experience [24]. The process is informal and relies on the intuition and judgment of the decision-maker. The environments where this decision-making process is most frequently used are those with shifting goals due to dynamic and changing conditions, time constraints and high stakes. Typically identified in firefighters or military personnel, this framework also appears to apply to CCC staff. Their decision-making relies heavily on using their judgment and experience to make decisions and modify plans to meet the needs of the situation in a workable and timely fashion [24,25].

Previous research has concluded that control measures in the form of standard guidance, education and hygiene are necessary to assist with the prevention and control of infectious diseases [26]. Other studies in areas of infection control and hygiene have highlighted issues related to compliance with guidance. To minimize these issues and increase compliance, they recommend guidance should be easy to follow, accessible and that identified challenges should be considered during their development. Strategies for changing practices should address needs at the individual and group level [27-29]. The staff in this study also highlighted these as considerations, and based on the findings in this study a number of factors were identified that could strengthen and be considered when developing further guidance to ensure optimal compliance.

### Consistent guidance

The process of identifying, managing and preventing enteric illness in children in CCC settings is inherently variable due to the number of factors, but continuing to enhance consistent decision-making tools and resources for all CCC staff is important. For example, consistently updating public health manuals and onsite visits from public health officials would be beneficial. A visual framework to assist decision-making, which could be used by CCC staff and parents, could be designed to include the variables identified by CCC staff in this study, as well as additional factors considered important by public health officials (e.g., blood in the stool). This framework could be designed to allow CCC staff to incorporate their experience and knowledge into it.

Decisions and actions rely on a clear and consistent understanding of "diarrhea" and "outbreak" and CCC staff indicated a need for this guidance. Developing one definition may not be possible when there are a number of factors to consider, but inclusion of these factors in a clear decision making framework could be of assistance to staff. It is important for public health officials and management of CCCs to work together while continuing to develop and strengthen definitions of diarrhea and outbreak. As well, it remains an important task to continue to clarify procedures and activities with all CCC staff to ensure consistency.

### Practical tools

In CCCs, staff require resources that they can use and adapt as needed. For example, improved record-keeping forms could be developed that are visual and easy for all staff to complete and which could improve challenges associated with time. The information collected on the forms is invaluable in illness surveillance for the entire centre. Staff were very aware of the children they work with but enhancing awareness and communication among staff could ensure the rapid implementation of preventative measures such as increased cleaning throughout the entire centre. This could be accomplished by exchanging information on a regular basis with staff in a consistent manner, through a regularly scheduled briefing session or standard communication log. Staff overwhelmingly expressed the desire for tools that would make the process of cleaning easier. This information is useful for public health officials and CCC management to consider when developing guidelines or providing guidance on cleaning methods and products that staff could use which are of high quality and efficiency but not seen as a challenge due to cost or time.

### Education

Consultation with public health officials indicated that public health training for CCC staff would be the most important tool to assist staff. In contrast, although continuing education offered by public health officials was important, CCC staff felt that education and information should also be made available to parents. This training would increase the parent's understanding of enteric disease and provide information on topics such as symptoms, the importance of exclusion and proper prevention and control. Outbreaks may provide educational opportunities to bring staff and parents together for education and information by public health officials. Therefore, it is suggested that educational material be directed to parents, as well as CCC staff.

### Bigger Picture: Policy and Procedures

Staff providing care to children on a daily basis were proud of the impact they had on children's development and education. The intrinsic value placed on child care is significant, but staff often felt that their work was undervalued. Staff need to be acknowledged for the work they do. Basic personal needs such as salaries, and paid sick days, should reflect the level of work and responsibility. Staff should not be penalized financially for taking time off when sick. Many of the staff relied on their wages to support themselves and their families and if not paid while ill, they might need to continue to work. Likewise, parents who keep their children home while ill receive no compensation for doing so, and in most cases they still pay for the days of care, even when their child is not there. Accommodations and incentives also need to be considered for families and parents, especially those in situations of financial need. In addition to staff specific needs, centres may require assistance to ensure additional staff for proper ratios, enhanced cleaning and ensuring substitute staff are available.

### Relationship with Public Health

The relationship between CCC staff in this study and local public health officials was very positive. During an outbreak, staff looked to public health officials to provide assistance and to reassure them that they took appropriate actions, particularly when dealing with parents. Staff stated a number of times that they referred parents to public health for further support and regarded public health officials as an authority figure when further assistance was required. Guidance should be supported by all groups involved. The relationship between staff, parents and public health is essential to ensuring proper response and management of enteric illness.

As with all qualitative research, questions regarding representativeness and generalizability must be addressed. This study was restricted to a small geographic area in one province in Canada. Although the data and recommendations appear applicable to other jurisdictions, it would be useful to hold similar groups with staff in other jurisdictions to explore and confirm these themes further, taking potential regional differences into account. The one group that was mixed with staff from different centres demonstrated the highest level of interaction from the participants and also gave the greatest degree of contrast. Further research should consider maximizing the number of mixed groups to gain further interaction and insight which would allow for a deeper comparison between groups in analysis due to the potential for contrasting discussion. The relationship between staff and parents is important as it relates to monitoring and preventing enteric illness in CCCs. Recommendations from this study will have impact on parents and therefore further research with parents is required before any recommendations should be implemented. An intervention study could be conducted to test the effectiveness of the recommendations in reducing the amount of illness or improving the response and understanding of staff. Further work with public health officials to gain their perspectives regarding strategies to implement recommendations would also be of value.

## Conclusion

This qualitative assessment provides an enhanced understanding and appreciation of the perspective, practices and challenges that staff of CCCs experience in responding to enteric illness and outbreaks. In general, it was found that CCC staff are dedicated to and well informed about the children they work with and have a tremendous responsibility. Results from this study will be useful to public health officials responsible for developing tools and resources to further support or better inform current knowledge and practices for preventing and managing enteric disease. The recommendations from this study were made based on data directly from staff of CCCs and are designed to be practical and developed in further collaboration with them. The experience and knowledge CCC staff use to identify and take action for prevention and management of enteric illness clearly demonstrates their responsibility as gatekeepers of health among the children they care for.

## Competing interests

The authors declare that they have no competing interests.

## Authors' contributions

MT designed the study, moderated the focus groups, collected and analyzed the data, and drafted the manuscript. CLA and AE participated in and provided critical feedback on the study design, the analyses and interpretation of results, as well as editorial comments on the manuscript. All authors read and approved the final manuscript

## Pre-publication history

The pre-publication history for this paper can be accessed here:



## References

[B1] Shonkoff J, Phillips D (2000). From Neurons to Neighbourhoods The Science of Early Childhood Development.

[B2] Early Childhood Education and Care as a Determinant of Health. http://www.phac-aspc.gc.ca/ph-sp/phdd/overview_implications/07_ecec.html.

[B3] Bushnik T (2006). Child Care in Canada: Children and Youth Research Paper Series. Statistics Canada.

[B4] CBC News Indepth: Daycare in Canada. http://www.cbc.ca/news/background/daycare/daycarecosts.html.

[B5] Doherty G, Lero DS, Goelman H, LaGrange A, Tougas J (2000). You Bet I care Study, A Canada Wide Study on: Wages, Working Conditions and Practices in Child Care Centres. Centre for Families, Work, and Well-Being, University of Guelph, Ontario.

[B6] O'Donnell JM, Thornton L, McNamara EB, Prendergrast T, Igoe D, Cosgrove C (2002). Outbreak of Vero cytotoxin-producing *Escherichia coli *O157 in a child care facility. Communicable Disease and Public Health.

[B7] Spika JS, Parsons JE, Nordenburg D, Wells JG, Gunn RA, Blake PA (1986). Hemolytic uremic syndromes and diarrhea associated with *Escherichia coli *O157:H7 in a day care centre. Journal of pediatrics.

[B8] Belongia EA, Osterholm MT, Soler JT, Ammend DA, Braun JE, MacDonald KL (1993). Transmission of *Escherichia coli *O157:H7 infection in Monnesota day-care facilities. Journal of the American Medical Association.

[B9] Galanis E, Longmore K, Hasselback P, Swann D, Ellis A, Panaro L (2003). Investigation of an *E. coli *O157:H7 outbreak in Brooks, Alberta, June-July 2002: the role of occult cases in the spread of infection within a daycare setting. Canada Communicable Disease Report.

[B10] Williams LD, Hamilton PS, Wilson BW, Estock MD (1997). An outbreak of *Escherichia coli *O157:H7 Involving Long Term Shedding and Person-to-Person Transmission in a Child Care Center. Environmental Health.

[B11] Gilbert M, Monk C, Wang H-L, Diplock K, Landry L Screening policies for daycare attendees: Lessons learned from an outbreak of *E. coli *O157:H7 in a daycare in Waterloo, Ontario. Canadian Journal of Public Health.

[B12] (2002). Report on the Review of the Approaches and Processes Utilized in the Investigation of an *Escherichia coli *O157:H7 outbreak in New Brunswick, December 2001. Presented to the Minister of Health and Wellness New Brunswick.

[B13] Hanks CA (2006). Community Empowerment: A Partnership Approach to Public Health Program Implementation. Policy, Politics, & Nursing Practice.

[B14] Mayan M (2001). An Introduction to Qualitative Methods: A training Module for Students and Professionals.

[B15] Krueger RA (1994). Focus Groups: A Practical Guide for Applied Research.

[B16] NVivo V 7.0. QSR International.

[B17] Last JM (2001). A Dictionary of Epidemiology.

[B18] Joseph HA, Shrestha-Kuwahara R, Lowry D (2004). Factors Influencing Health Care Workers' Adherence to Work Site Tuberculosis Screening and Treatment Policies. American Journal of Infection Control.

[B19] Chan EA, Chung JW, Wong TK (2007). Learning from the severe acute respiratory syndrome (SARS) epidemic. Clinical Nursing Research.

[B20] Lymer UB (2003). Health care workers' action strategies in situation that involve a risk of blood exposure. Journal of Clinical Nursing.

[B21] Lipsky M (1983). Street level bureaucracy-Dilemmas of the individual in public service.

[B22] Cioffi J (2000). A study of the use of past experiences in clinical decision making in emergency situations. International Journal of Nursing Studies.

[B23] Cioffi J (2000). Recognition of patients who require emergency assistance: A descriptive study. Heart and Lung.

[B24] Klein G, Klinger D (1991). Naturalistic Decision Making. Human Systems IAC Gateway Volume XI.

[B25] Bond S, Cooper S (2006). Modelling emergency decisions: recognition-primed decision making. The literature in relation to an ophthalmic critical incident. Journal of Clinical Nursing.

[B26] Nesti MM, Goldbaum M (2007). Infectious diseases and daycare and preschool education. Jornal de Petiatria.

[B27] Lymer UB, Richt B, Isaksson B (2004). Blood exposure: factors promoting health care workers' compliance with guidelines in connection with risk. Journal of Clinical Nursing.

[B28] Novoa AM, Pi-Sunyer T, Sala M, Molins E, Castells X (2007). Evaluation of hand hygiene adherence in a tertiary hospital. American Journal of Infection Control.

[B29] Creedon SA (2005). Healthcare workers' hand decontamination practices: compliance with recommended guidelines. Journal of Advanced Nursing.

